# Impact of a Recipe Kit Scheme (BRITE Box) on Cooking and Food‐Related Behaviours of Children and Families: Exploring Parental/Carer Views

**DOI:** 10.1111/jhn.70038

**Published:** 2025-03-13

**Authors:** Sarah Sumpter, Ruth Dawson, Nick Dawson, Nevena Nancheva, Ronald Ranta, Dee Bhakta, Hilda Mulrooney

**Affiliations:** ^1^ School of Life Sciences, Pharmacy & Chemistry, Kingston University London UK; ^2^ Voices of Hope Kingston London UK; ^3^ School of Arts, Humanities and Social Sciences, University of Roehampton London UK; ^4^ School of Law, Social and Behavioural Sciences, Kingston University London UK; ^5^ School of Human Sciences, London Metropolitan University London UK

**Keywords:** confidence, cooking, diet, diversity, family, meal kit, skills

## Abstract

**Background:**

Dietary intakes in UK children fail to meet national recommendations, especially in low‐income groups. Involving children in food preparation and cooking may enhance acceptability of a wider range of foods, enhance their skills and increase their enjoyment of food. An innovative recipe meal kit scheme, Building Resilience in Today's Environment (BRITE) Box, was developed during the pandemic primarily to address food insecurity (FI). Administered via schools, it offers pre‐weighed ingredients sufficient for a meal for a family of five, plus a child‐focused recipe, weekly during school termtimes.

**Methods:**

Qualitative and quantitative exploration of BRITE Box using questionnaires and semi‐structured interviews among parents/carers of children receiving the boxes was conducted at two timepoints a year apart.

**Results:**

A total of 154 parents/carers completed questionnaires and 29 were interviewed. Responses indicated multiple benefits of the scheme, including increased confidence in cooking among both children and parents/carers. Both questionnaire responses and interviews suggested improvements in a range of food‐related behaviours, including cooking and eating together and talking more about food. Parents/carers suggested that their children were more willing to eat vegetables and healthy foods and to try new foods and flavours. They also reported greater use of leftovers thereby potentially reducing food waste. Improved behaviours, willingness to try new foods and flavours, reduced food waste and lower stress of trying to think of new and acceptable family meals are likely to have contributed to the positive impact on their mental health reported by BRITE Box parents/carers.

**Conclusions:**

Meal kits for children may improve dietary diversity, enhance enjoyment and skills and impact positively on a range of family food‐related behaviours. We argue that BRITE Box has the potential for widespread positive impacts on cooking and food‐related behaviours in children and families, meriting wider study and dissemination as a positive approach to healthy eating in children.

## Summary

- Meal kits devised specifically for children can increase their acceptance of a wider range of foods, and their engagement with food preparation and cooking.
- In addition, they can benefit wider family food behaviours including eating and cooking together.

## Graphical abstract

Parent/carer views of a recipe box scheme for children providing free, weekly, pre‐weighed ingredients suggest their potential for improving diets, increasing skills (e.g., cooking, reading and measuring) and enhancing family resilience. Boxes evoked happiness and excitement, children gained confidence and and families cooked, talked about food and ate together more.

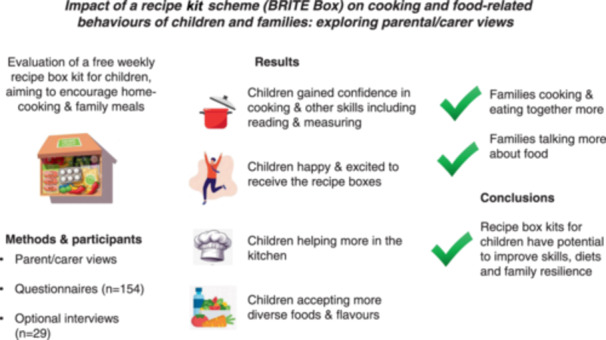

## 1 Introduction

Food insecurity (FI), poor diets and dietary ill health are major causes of concern for UK children and families. Through exploring parental perspectives, this article examines the impact of Building Resilience in Today's Environment (BRITE Box), an innovative recipe meal kit scheme, on cooking and food‐related behaviours of children and families.

FI affected an estimated 14.8% of UK households in January 2024 [1], having increased during the pandemic and subsequent cost‐of‐living crisis [2]. It describes lack of regular access to adequate safe and nutritious food to enable health and optimal growth and development [3]. FI affects some groups more than others; one such group being families with children—approximately one quarter of families with children are food insecure [1], and it is approximately four times more common in households receiving Universal Credit (means‐tested benefits) than not (e.g., 41.9% vs. 10.6% in June 2024) [1]. Those struggling with FI tend to have less nutritious diets [4, 5, 6], affecting long‐term health since diet is a major modifiable risk factor for chronic disease [7]. Poor diets are a longstanding problem in the United Kingdom. Primary school children aged 4–10 years have inadequate intakes of fibre, fruit and vegetables, whereas consumption of free sugars, sugar‐sweetened beverages (SSBs) and saturated fat exceed recommendations despite recent falls in free sugar and SSB intakes [6]. Per calorie, the cost of healthy foods is approximately double that of less healthy foods [8], and food prices rose by an estimated 30.6% between May 2021 and May 2024 [9]. Given this, it is unsurprising that children's diets are not ideal, but it is concerning given the importance of nutritious diets for their growth and development [10].

Among low‐income groups, food represents a greater proportion of their budgets, leaving them less scope to navigate food price rises [2, 11]. Managing on low incomes requires a range of complex skills including budgeting, identifying low‐cost healthy options and using leftovers [12]. Some suggest that FI individuals have lower levels of these skills [13, 14, 15], and therefore education is a viable approach to addressing FI. Others suggest that there is little or no difference in skills by income [16, 17, 18, 19], and lack of finance [20, 21, 22, 23, 24] rather than lack of skills drives FI. Family meals may represent a way of mitigating both FI and poor diets in children since frequency of family meals is associated with improved nutritional quality and healthy diet in children (< 11 years) [25, 26] and adolescents (> 11 years) [26], more positive attitudes towards family meals and better mealtime environments [27]. Despite this, food preparation requires time and effort [28], more difficult for time‐poor households potentially navigating long working hours [29] and/or shiftwork [30]. Lower income households are more likely to be time‐poor [31] and food insecure [32]. The development of cooking skills in children may encourage better nutrition [33], and parents support the development of such skills in their children [34, 35] but cite both time and fear as barriers [35]. Encouraging cooking together and family meals without additional burden for already time‐poor households has potential to improve nutrition and skills acquisition in children.

Building resilience in children is also an issue. The adverse impact of the pandemic and successive lockdowns on social, academic skills and mental health of children is evident. Childhood mental health problems spiked in the pandemic but increased over the last two decades and were associated with worse educational outcomes in a longitudinal study of 5–16‐year‐olds in the United Kingdom [36]. In 2023, an estimated 20.3% of children and young people (aged 8–16 years) had a probable mental health disorder; higher among those who could not afford extracurricular activities [37]. A bidirectional relationship between FI and poor mental health has been demonstrated [38]; those with mental health conditions being more than twice as likely as those without to struggle with FI (28.0% vs. 10.7%, respectively), whereas FI itself imposes a psychological strain adversely affecting mental health and well‐being, including among children [38]. Supporting children to develop skills and increase their resilience therefore has potential benefits across multiple aspects of their lives including educational, social and mental well‐being.

## 2 BRITE Box: Context

BRITE Box is a recipe meal kit initiative specifically designed for primary school‐aged children, providing pre‐weighed ingredients and a recipe card weekly during termtime via schools (each term approximately 12 weeks excluding weekends and holidays). Ingredients are sufficient to make a meal for a family of five, and replication of the meals costs < £5. It was initially established in April 2020 as a crisis response to FI in children but extends beyond that to encourage children and families to cook and eat a healthy meal together, thereby increasing child and family resilience and encouraging adoption and maintenance of health‐promoting behaviours such as cooking and eating together. Provided free to children and families (usually for one academic year: three terms consisting of 12 weeks each), it is funded by grants (e.g., from local authorities) and community fundraising efforts. Boxes are prepared by volunteers and distributed by schools. Schools have wide latitude over which children receive them; FI is a major driver, but children can receive them for other reasons (e.g., very limited dietary intakes). BRITE Box recipes focus on vegetables and are designed to be healthy and possible for children to make (with adult supervision). Most are savoury; an additional fruit‐based dessert recipe is included termly. Separately, nutritional analysis of the recipes has been conducted (not reported here), and they have been adjusted in line with increasing ingredient costs and to enhance their sustainability.

Here we describe parental/carer perceptions of the effect of BRITE Box on cooking and food‐related behaviours of children and families as part of an ongoing iterative evaluation of the initiative.

## 3 Methods

BRITE Box was evaluated twice, using questionnaires and optional interviews to collect qualitative and quantitative data from parents/carers, volunteers, organisers, funders and suppliers. The primary purpose of the evaluation was to evidence the impact of the initiative and identify areas for improvement and innovation. Ethics approval for the evaluation was granted by Kingston University Faculty Ethics Committee. Only the views of parents/carers are reported here.

### 3.1 Data Tools

All data tools were codeveloped with the BRITE Box organisers (R.D. & N.D.) to ensure they were fit‐for‐purpose; however, the evaluation was conducted entirely independently by the research team (S.S., R.R., N.N., D.B. and H.M.M.). Questionnaires for parents/carers were constructed online using Microsoft Forms. Limited demographics data including age, gender, ethnicity, disability status and family size, identified from the literature [1] as likely to affect experiences of FI, were collected. In relation to BRITE Box itself, the organisers identified the key questions requiring exploration and the research team constructed bespoke questionnaires to evaluate the service provision. Participants were asked to rate a series of statements exploring its impact on food‐related behaviours (e.g., cooking, eating together); impact on children and the family (e.g., trying new foods, increased confidence in cooking) and skills acquisition of children using a five‐point Likert rating scale (from ‘strongly agree’ to ‘strongly disagree’). They were also asked to articulate what they liked most and least and their favourite recipes using open text boxes. The final questionnaire is shown in Supporting Information Appendix 1; this was agreed by the organisers and the research team after multiple iterations. It was not piloted; the same questionnaire was used at both timepoints (TPs).

Optional semi‐structured interviews to further explore parental/carer perceptions were held by telephone with those willing to do so. A qualitative descriptive approach was adopted [39, 40] based on naturalistic inquiry [41, 42] that is placing the observations made within the context of participants’ lives, without seeking to change or manipulate them.

### 3.2 Data Collection

i. Questionnaires:
  Invitations were distributed in recipe boxes over several weeks using a flier with a QR code to the online questionnaire in Microsoft Forms. Questionnaires included an invitation to participate in optional interviews; those who left their contact details were subsequently contacted (otherwise completion was anonymous). Participant information sheets and consent forms were embedded within the questionnaires, which could not be completed unless explicit consent was agreed.
ii. Interviews:
  Interviews were organised and conducted by three members of the research team online or by telephone (R.R., N.N. and H.M.M.). Interview guides, with questions developed by the organisers and research team to explore parents/carers’ experiences of the scheme in greater detail, were used to ensure consistency (Supporting Information Appendix 2). Interviews were audio recorded for accuracy and additional contemporaneous notes taken and used to sense‐check the meanings of the transcripts. Interviewees received a small token of acknowledgement of their time in the form of a £10 Amazon voucher. Interviews lasted on average 19.24 ± 13.54 min. The research team, composed of social scientists and nutritionists, have previous experience of researching issues concerning FI, and are and have been involved as volunteers, coordinators and trustees, in a number of different schemes, including food banks.
  Data were collected at two TPs from parents/carers of children then receiving boxes. Participation in the scheme is for one academic year; as the evaluations were conducted 1 year apart it is unlikely that parents/carers at TP1 also participated at TP2. However, it is possible if more than one child attended the school. TP1 was May–September 2022 and TP2 was May–September 2023. In 2022, 285 children at 21 schools received BRITE Box: respectively 368 and 33 in 2023. All those receiving the boxes were eligible to complete both questionnaires and interviews.

### 3.3 Data Analysis

i. Questionnaires:
  Questionnaires were coded and data entered manually into an Excel spreadsheet. Statistical analysis was conducted using IBM SPSS version 27. Differences in levels of agreement with statements by demographic characteristics were assessed using Kruskal–Wallis tests with post hoc Dunn's and Bonferroni correction. Differences in responses between TP were tested using Mann–Whitney *U* tests at *p* < 0.05. For similar statements, levels of reliability were tested using Cronbach's analysis. Similar statements are those shown in Tables 3 (excluding ‘My child/ren are embarrassed by getting BRITE Box’) and 4, 5, 6. High levels of reliability were found, with a Cronbach's alpha of 0.96. Responses within open text boxes were collated and analysed qualitatively, similarly to the interview responses (see below).
ii. Interviews:
  Audio recordings were transcribed verbatim, and basic thematic analysis was carried out to identify key themes [43]. Thematic analysis was carried out separately by three members of the research team (H.M.M., R.R. and N.N.), taking an inductive approach. They listened on multiple occasions to the audio recordings, using those and the transcripts to identify themes and subthemes in an iterative process, manually coding, reviewing and agreeing them [43]. Since BRITE Box aims to change behaviours, the themes were then mapped against the commonly used COM‐B model of behaviour change [44], to identify whether they related to capability, opportunity or motivational aspects of behaviour change (the model was not used to identify themes, but simply to ascertain which aspect of this model the identified themes related to). Any verbatim quotes used to demonstrate themes have been anonymised.

## 4 Results

### 4.1 Questionnaires

A total of 82 parents/carers completed questionnaires at TP1 and 72 at TP2, therefore a total of 154 parents/carers are included. The majority of questionnaire respondents at both TP were women and aged 30–49 years. Just over two‐thirds were White (Table 1). Approximately 15% at both TP self‐identified as having a disability. There were no significant differences in age (*p* = 0.32), gender (*p* = 0.78), ethnicity (*p* = 0.17) or disability (*p* = 0.59) between TP.

**Table 1:** Age, gender and ethnicity characteristics of BRITE Box users. Data are expressed as numbers (%).

Age (years)													
	< 30			30–39			40–49			50–59		≥ 60	Differences by TP
TP1 (*n* = 82)	7 (8.5)			33 (40.2)			27 (32.9)			15 (18.3)		0 (0.0)	*Z* = −1.002, *p* = 0.32
TP2 (*n* = 71)^a^	9 (12.5)			26 (36.1)			34 (47.2)			2 (2.8)		1 (1.4)	
Total (*n* = 153)^a^	16 (10.4)			59 (38.3)			61 (39.6)			17 (11.0)		1 (0.6)	

Gender													
			Woman					Man					
TP1 (*n* = 82)			75 (91.5)					7 (8.5)					*Z* = −0.28, *p* = 0.78
TP2 (*n* = 71)^a^			65 (91.5)					6 (8.5)					
Total (*n* = 153)			140 (91.5)					13 (8.5)					

Ethnicity													
		White			Black	Asian			Mixed		Other		
TP1 (*n* = 79)^a^		57 (72.2)			7 (8.9)	9 (11.4)			2 (2.5)		4 (5.1)		*Z* = −1.39, *p* = 0.17
TP2 (*n* = 68)^a^		44 (64.7)			5 (7.4)	7 (10.3)			2 (2.9)		10 (14.7)		
Total (*n* = 147)^a^		101 (68.7)			12 (8.2)	16 (10.9)			4 (2.7)		14 (9.5)		

Do you consider yourself to have a disability?													
				**Yes**						**No**			
TP1 (*n* = 81)^a^				12 (14.8)						69 (85.2)			*Z* = −0.59, *p* = 0.59
TP2 (*n* = 69)^a^				10 (14.5)						59 (85.5)			
Total (*n* = 150)^a^				22 (14.7)						128 (85.3)			

a Not all participants answered all questions. Calculations are based on the numbers who did, and this is shown for each question.

Approximately a third of respondents had two children; however, approximately 13%–15% at both TPs had four children (Table 2). All of the children involved in the scheme were aged 5–11 years. Almost half had received boxes for at least 6 months. There were no significant differences in number of children (*p* = 0.86) or duration receiving boxes (*p* = 0.29), by TP.

**Table 2:** Number of children and duration receiving BRITE Box from questionnaire and interview participants. Data are expressed as numbers (%).

Number of children (questionnaire respondents)												
	1		2		3		4		5		≥ 6	Differences by TP
TP1 (*n* = 82)	20 (24.4)		28 (34.1)		18 (22.0)		11 (13.4)		3 (3.7)		2 (2.4)	*p* = 0.86
TP2 (*n* = 72)	20 (27.8)		23 (31.9)		13 (18.1)		11 (15.3)		4 (5.6)		1 (1.4)	
Total (*n* = 154)	40 (26.0)		51 (33.1)		31 (20.1)		22 (14.3)		7 (4.5)		3 (1.9)	

Duration of BRITE Box (months; questionnaire respondents)												
	**0–3**				**3–6**				**> 6**			
TP1 (*n* = 80)^a^	18 (22.5)				24 (30.0)				38 (47.5)			*p* = 0.29
TP2 (*n* = 72)	28 (38.9)				13 (18.1)				31 (43.1)			
Total (*n* = 152)^a^	46 (30.3)				37 (24.3)				69 (45.4)			

Number of children (interview respondents)												
	**1**	**2**		**3**		**4**		**5**		**≥ 6**		
TP1 (*n* = 15)	3 (20.0)	5 (33.3)		4 (26.7)		2 (13.3)		0 (0.0)		1 (6.7)		*p* = 0.52
TP2 (*n* = 14)	7 (50.0)	3 (21.4)		1 (7.1)		1 (7.1)		1 (7.1)		1 (7.1)		
Total (*n* = 29)	10 (34.5)	8 (27.6)		5 (17.2)		3 (10.3)		1 (3.4)		2 (6.9)		

Duration of BRITE Box (months; interview respondents)												
	0–3				3–6				> 6			
TP1 (*n* = 15)	4 (26.7)				4 (26.7)				7 (46.7)			*p* < 0.05
TP2 (*n* = 14)	7 (50.0)				3 (21.4)				4 (28.6)			
Total (*n* = 29)	11 (37.9)				7 (24.1)				11 (37.9)			

a Not all participants answered all questions. Calculations are based on the numbers who did, and this is shown for each question.

BRITE Box was very positively received; 81.4% of parents/carers agreed or strongly agreed that their child was excited to receive the box.

By contrast, 77.4% disagreed or strongly disagreed that their child was embarrassed to receive BRITE Box (Table 3). Children gained confidence from BRITE Box; 76.2% agreed or strongly agreed that their child had gained confidence with food and cooking since starting BRITE Box.

**Table 3:** Responses to emotion and confidence statements in relation to BRITE Box. Data are expressed as numbers (%).

My child/ren are excited when we get a BRITE Box						
	Strongly agree	Agree	Neither agree nor disagree	Disagree	Strongly disagree	Differences by TP
TP1 (*n* = 82)	44 (53.7)	24 (29.3)	9 (11.0)	1 (1.2)	4 (4.9)	*p* = 0.58
TP2 (*n* = 72)	45 (62.5)	12 (16.7)	6 (8.3)	1 (1.4)	8 (11.1)	
Total (*n* = 154)	89 (57.8)	36 (23.4)	15 (9.7)	2 (1.3)	12 (7.8)	

I am more confident in the kitchen since starting BRITE Box						
	**Strongly agree**	**Agree**	**Neither agree nor disagree**	**Disagree**	**Strongly disagree**	
TP1 (*n* = 78)^a^	23 (29.5)	17 (21.8)	24 (30.8)	9 (11.5)	5 (6.4)	*p* = 0.03
TP2 (*n* = 72)	23 (31.9)	18 (25.0)	20 (27.8)	4 (5.6)	7 (9.7)	
Total (*n* = 150)^a^	46 (30.7)	35 (23.3)	44 (29.3)	13 (8.7)	12 (8.0)	

My child/ren are more confident about food/cooking since starting BRITE Box						
	**Strongly agree**	**Agree**	**Neither agree nor disagree**	**Disagree**	**Strongly disagree**	
TP1 (*n* = 79)^a^	39 (49.4)	23 (29.1)	11 (13.9)	4 (5.1)	2 (2.5)	*p* = 0.91
TP2 (*n* = 72)	35 (48.6)	18 (25.0)	14 (19.4)	1 (1.4)	4 (5.6)	
Total (*n* = 151)^a^	74 (49.0)	41 (27.2)	25 (16.6)	5 (3.3)	6 (4.0)	

My child/ren are embarrassed by getting BRITE Box						
	**Strongly agree**	**Agree**	**Neither agree nor disagree**	**Disagree**	**Strongly disagree**	
TP1 (*n* = 74)^a^	1 (1.4)	1 (1.4)	9 (12.2)	18 (24.3)	45 (60.8)	*p* < 0.001
TP2 (*n* = 72)	5 (6.9)	2 (2.8)	15 (20.8)	24 (33.3)	26 (36.1)	
Total (*n* = 146)^a^	6 (4.1)	3 (2.1)	24 (16.4)	42 (28.8)	71 (48.6)	

a Not all participants answered all questions. Calculations are based on the numbers who did, and this is shown for each question.

Just over half (54.0%) of parents/carers felt that they themselves had gained more confidence in the kitchen since starting BRITE Box, however, almost a third (29.3%) were equivocal.

Parent/carers’ gender, ethnicity, age and disability were not associated with their child's excitement, embarrassment or confidence in food and/or cooking (data not shown). However, ethnicity was associated with the extent to which parents/carers gained confidence in cooking. White parents were significantly less likely to agree or strongly agree that they themselves had gained confidence in the kitchen since starting BRITE Box, compared with Asian parents (47.5% vs. 87.5%, *p* < 0.01). There were no significant differences in responses by TP (Table 3), with the exception of embarrassment—significantly more parents/carers disagreed or strongly disagreed that their child was embarrassed at TP1 than 2 (85.1% vs. 69.4%, respectively; *p* < 0.001). No impact of duration or number of children was found (data not shown).

BRITE Box introduced recipients to new foods and flavours. Most parents/carers (81.4%) agreed or strongly agreed that their children tried new foods because they were involved in preparation or cooking, whereas 80.5% agreed or strongly agreed that their family had tried new foods since starting BRITE Box. In the case of new flavours and new and different kitchen skills, 78.8% and 71.8%, respectively, agreed or strongly agreed. Responses did not differ by TP (Table 4). No difference in responses by demographic factors such as age, gender, ethnicity, number of children and disability status was observed, and BRITE Box duration had no impact (data not shown).

**Table 4:** Impact of BRITE Box on new food‐related behaviours. Data are expressed as numbers (%).

We have tried new foods since starting BRITE Box						
	Strongly agree	Agree	Neither agree nor disagree	Disagree	Strongly disagree	Differences by TP
TP1 (*n* = 77)^a^	42 (54.5)	24 (31.2)	6 (7.8)	4 (5.2)	1 (1.3)	*p* = 0.47
TP2 (*n* = 72)	44 (61.1)	10 (13.9)	8 (11.1)	3 (4.2)	7 (9.7)	
Total (*n* = 149)^a^	86 (57.7)	34 (22.8)	14 (9.4)	7 (4.7)	8 (5.4)	

BRITE Box has introduced us to new flavours						
	**Strongly agree**	**Agree**	**Neither agree nor disagree**	**Disagree**	**Strongly disagree**	
TP1 (*n* = 74)^a^	43 (58.1)	21 (28.4)	3 (4.1)	4 (5.4)	3 (4.1)	*p* = 0.91
TP2 (*n* = 72)	39 (54.2)	12 (16.7)	11 (15.3)	1 (1.4)	9 (12.5)	
Total (*n* = 146)^a^	82 (56.2)	33 (22.6)	14 (9.6)	5 (3.4)	12 (7.8)	

We have learnt new and different kitchen skills because of BRITE Box						
	**Strongly agree**	**Agree**	**Neither agree nor disagree**	**Disagree**	**Strongly disagree**	
TP1 (*n* = 77)^a^	30 (39.0)	24 (31.2)	12 (15.6)	10 (13.0)	1 (1.3)	*p* = 0.09
TP2 (*n* = 72)	36 (50.0)	17 (23.6)	12 (16.7)	1 (1.4)	6 (8.3)	
Total (*n* = 149)^a^	66 (44.3)	41 (27.5)	24 (16.1)	11 (7.4)	7 (4.7)	

My child/ren have tried new foods because they have been involved in preparing/cooking them						
	**Strongly agree**	**Agree**	**Neither agree nor disagree**	**Disagree**	**Strongly disagree**	
TP1 (*n* = 79)^a^	40 (50.6)	29 (36.7)	5 (6.3)	3 (3.8)	2 (2.5)	*p* = 0.33
TP2 (*n* = 72)	31 (43.1)	23 (31.9)	12 (16.7)	0 (0.0)	6 (8.3)	
Total (*n* = 154)^a^	71 (47.0)	52 (34.4)	17 (11.3)	3 (2.0)	8 (5.3)	

a Not all participants answered all questions. Calculations are based on the numbers who did, and this is shown for each question.

Recipients agreed or strongly agreed that several positive family food‐related behaviours were increased by participation in BRITE Box. These included cooking together, eating together, using leftovers, eating healthy foods, vegetable consumption and talking about food (Table 5). There was little difference by demographic factors, BRITE Box duration, TP or number of children. However, significantly more respondents used leftovers at TP2 than TP1 (65.3% vs. 47.5%, *p* = 0.047). Ethnicity also affected use of leftovers; significantly more Asian than White participants agreed or strongly agreed that they used more leftovers because of BRITE Box (81.2% vs. 49.5%, *p* = 0.048). Other differences were also found. Significantly more Asian than White participants reported talking more about food (100% vs. 68.8%, respectively, *p* = 0.012) and eating more healthy foods since starting BRITE Box (87.5% vs. 56.4%, respectively, *p* = 0.002).

**Table 5:** Impact of BRITE Box on family food‐related behaviours. Data are expressed as numbers (%).

We have cooked together more because of BRITE Box						
	Strongly agree	Agree	Neither agree nor disagree	Disagree	Strongly disagree	Differences by TP
TP1 (*n* = 78)^a^	44 (56.4)	20 (25.6)	6 (7.7)	7 (9.0)	1 (1.3)	*p* = 0.65
TP2 (*n* = 72)	34 (47.2)	22 (30.6)	10 (13.9)	1 (1.4)	5 (6.9)	
Total (*n* = 150)^a^	78 (52.0)	42 (28.0)	16 (10.7)	8 (5.3)	6 (4.0)	

We eat together as a family more since we started BRITE Box						
	**Strongly agree**	**Agree**	**Neither agree nor disagree**	**Disagree**	**Strongly disagree**	
TP1 (*n* = 79)^a^	25 (31.6)	18 (22.8)	20 (25.3)	12 (15.2)	4 (5.1)	*p* = 0.55
TP2 (*n* = 72)	25 (34.7)	15 (20.8)	18 (25.0)	8 (11.1)	6 (8.3)	
Total (*n* = 151)^a^	50 (33.1)	33 (21.9)	38 (25.2)	20 (13.2)	10 (6.6)	

We use more leftovers since starting BRITE Box						
	**Strongly agree**	**Agree**	**Neither agree nor disagree**	**Disagree**	**Strongly disagree**	
TP1 (*n* = 80)^a^	21 (26.3)	17 (21.3)	24 (30.0)	15 (18.8)	3 (3.8)	*p* < 0.05
TP2 (*n* = 72)	24 (33.3)	23 (31.9)	15 (20.8)	5 (6.9)	5 (6.9)	
Total (*n* = 152)^a^	45 (29.6)	40 (26.3)	39 (25.7)	20 (13.2)	8 (5.3)	

We eat more vegetables since starting BRITE Box						
	**Strongly agree**	**Agree**	**Neither agree nor disagree**	**Disagree**	**Strongly disagree**	
TP1 (*n* = 78)^a^	22 (28.2)	23 (29.5)	14 (17.9)	14 (17.9)	5 (6.4)	*p* = 0.14
TP2 (*n* = 72)	25 (34.7)	23 (31.9)	11 (15.3)	4 (5.6)	9 (12.5)	
Total (*n* = 150)^a^	47 (31.3)	46 (30.7)	25 (16.7)	18 (12.0)	14 (9.3)	

We eat more healthy foods because of BRITE Box						
	**Strongly agree**	**Agree**	**Neither agree nor disagree**	**Disagree**	**Strongly disagree**	
TP1 (*n* = 78)^a^	21 (26.0)	20 (25.6)	28 (35.9)	5 (6.4)	4 (5.1)	*p* = 0.71
TP2 (*n* = 72)	24 (33.3)	13 (18.1)	21 (29.2)	5 (6.9)	9 (12.5)	
Total (*n* = 150)^a^	45 (30.0)	33 (22.0)	49 (32.7)	10(6.7)	13 (8.7)	

We talk more about food since we started BRITE Box						
	**Strongly agree**	**Agree**	**Neither agree nor disagree**	**Disagree**	**Strongly disagree**	
TP1 (*n* = 77)^a^	20 (26.0)	28 (36.4)	15 (19.5)	11 (14.3)	3 (3.9)	*p* = 0.32
TP2 (*n* = 72)	25 (34.7)	17 (23.6)	18 (25.0)	7 (9.7)	5 (6.9)	
Total (*n* = 149)^a^	45 (30.2)	45 (30.2)	33 (22.1)	18 (12.1)	8 (5.4)	

a Not all participants answered all questions. Calculations are based on the numbers who did, and this is shown for each question.

BRITE Box recipes were popular; 74.0% agreed or strongly agreed that they always used them and 86.0% that they would use them again (data not shown). No effect of demographic factors, TP, number of children or length of time BRITE Box was received was found (data not shown).

The majority of participants (89.3%) had a positive experience of BRITE Box, and 74.5% agreed or strongly agreed that it helped with their food budgets. Two‐thirds agreed or strongly agreed that it benefited their mental health (Table 6), in part due to less pressure about food waste. No effects of demographic or other characteristics were found (data not shown).

**Table 6:** BRITE Box, overall experience and impact on mental health and well‐being and food budget. Data are expressed as numbers (%).

Overall, our experience of BRITE Box has been positive						
	Strongly agree	Agree	Neither agree nor disagree	Disagree	Strongly disagree	Differences by TP
TP1 (*n* = 78)^a^	0 (0.0)	1 (1.3)	4 (5.1)	10 (12.8)	63 (80.8)	*p* = 0.10
TP2 (*n* = 72)	8 (11.1)	0 (0.0)	3 (4.2)	5 (6.9)	56 (77.8)	
Total (*n* = 150)^a^	8 (5.3)	1 (0.7)	7 (4.7)	15 (10.0)	119 (79.3)	

Taking part in BRITE Box has improved our mental well‐being						
	**Strongly agree**	**Agree**	**Neither agree nor disagree**	**Disagree**	**Strongly disagree**	
TP1 (*n* = 78)^a^	24 (30.8)	27 (34.6)	23 (29.5)	1 (1.3)	3 (3.8)	*p* = 0.43
TP2 (*n* = 72)	26 (36.1)	22 (30.6)	16 (22.2)	2 (2.8)	6 (8.3)	
Total (*n* = 150)^a^	50 (33.3)	49 (32.7)	39 (26.0)	3 (2.0)	9 (6.0)	

BRITE Box helps me with my food budget						
	**Strongly agree**	**Agree**	**Neither agree nor disagree**	**Disagree**	**Strongly disagree**	
TP1 (*n* = 77)^a^	32 (41.6)	25 (32.5)	15 (19.5)	3 (3.9)	2 (2.6)	*p* = 0.17
TP2 (*n* = 72)	37 (51.4)	17 (23.6)	10 (13.9)	2 (2.8)	6 (8.3)	
Total (*n* = 149)^a^	69 (46.3)	42 (28.2)	25 (16.8)	5 (3.4)	8 (5.4)	

a Not all participants answered all questions. Calculations are based on the numbers who did, and this is shown for each question.

The statement with the highest level of agreement across both TP was that BRITE Box was a positive experience. Overall, 89.3% of respondents agreed or strongly agreed. This was followed by reusing the recipes and children trying new foods due to their involvement in cooking or preparing them (86.0% and 80.0% average agreement, respectively, data not shown).

Lowest levels of agreement were to the statement ‘My child/ren are embarrassed when we get a BRITE Box’ (6.2% average agreement) followed by ‘We never follow the BRITE Box recipe’ (24.0% average agreement, data not shown).

### 4.2 Interviews

A total of 29 interviews with parents/carers were held, 15 at TP1 and 14 at TP2. Over half at both TPs had 1‐2 children, with no difference by TP (*p *= 0.52). Significantly more parents interviewed at TP2 were receiving BRITE Box for 0–3 months (50.0% vs. 26.7%, respectively, *p* < 0.05). A major interview theme was help (with budget and meal ideas). Another was children's engagement and skills acquisition. This included cooking but also wider skills such as reading and maths (e.g., measurements). Positive emotions associated with the boxes were also highlighted including enthusiasm and excitement. Also frequently mentioned were the new foods and flavours tried by children and families (Table 7). In relation to behaviour change theory, the themes identified corresponded with the capability, opportunity and motivation elements of the COM‐B model [44]. The ‘Help’ theme (with subthemes of help with budgets, ideas for dishes and reuse of the recipes) related to **opportunity** by enabling access to foods and recipes, and by providing ingredients for a family meal, helped financially. The ‘child skills/engagement’ theme (with subthemes of skills acquisition, helping adults to cook and specific skills), related to **capability** within the model. ‘Positive emotions’ related to **motivation** within the model, whereas the ‘new/novel’ theme (with subthemes of variety, foods and recipes) linked to both **opportunity** and **capability** within the model. Both the ‘experience’ (positive experience increasing willingness to try new foods) and ‘togetherness’ themes linked to **motivation** in the COM‐B model [44].

**Table 7:** Main themes from qualitative analysis of interviews (*n* = 29 interviewees). Data are expressed as numbers.

Theme	Subthemes	Illustrative quotes using pseudonyms	COM‐B [43] attribute
Help (*n* = 68)	Budget (*n* = 29) Reuse of recipes (*n* = 21) Ideas for meals (*n* = 18)	‘On a low income you can become a bit stressy about eating, if they're not eating what you cook’ (mother of 3 children, TP1) ‘And, like, we sort of used the, like, the same meals on a certain night and they've actually helped by giving her a bit more variety’ (mother of 10‐year‐old SEN child) ‘But we keep them all, we put them [the recipes] in a little recipe book ourselves’ (mother of 9‐year‐old twins, TP1). ‘It helps me save more money, because I don't have to buy too much food, because I know that BRITE Box will also help with a meal’ (mother of 2 children, TP2) ‘I have kept them [the recipes] all because we can have a look over them and erm try them again’ (mother of 9‐year‐old, TP2) ‘I also enjoy the fact that on a Friday when it's the end of the week not having to think about what we're going to cook that's really a good thing. You know, it's [the ingredients] all provided. It's a treat’ (mother of 9‐year‐old, TP1)	Opportunity: Access allows trial without risk, reduces stress associated with meal planning and preparation
Child skills/engagement (*n* = 63)	New skills gained (*n* = 30) Helping (adult) with cooking (*n* = 21) Specific skills for example reading recipes, measuring, following instructions, knife skills (*n* = 12)	‘It's [the recipe] very child‐friendly…she says, “I can do it”’ (mother of 3 children, TP2) *‘*The claw bridge method. And he knows how to measure with a scale’ (mother of 2 children, TP2) ‘But I say to them like, “If you're gonna help in the kitchen you need to read the instructions, and they do. They sit there and they read it [the recipe] and they work out the time and how long we've got left and things like that”’ (mother of 9‐year‐old twins, TP1) ‘Because I am Indian I do not cook that much English food, I do not know this so much so I am learning as well you see, it's really good for the adult and for the children’ (mother of 7 children, TP2) ‘She'd be able to read a few of the words [in the recipe] – I would read them out to her and then follow the directions. So she knew or had a better understanding that it was a process and there were steps to getting or making and preparing the food. So yes, it helped with her reading most definitely’ (mother of 2 children, TP1) ‘I think she's learned a lot about meat hygiene. you know, making sure it's properly defrosted and washing your hands after you touch it [meat] and things like that. So I'm pleased because I think she's started putting some of the foundations down in how to cook’ (mother of 9‐year‐old SEN child, TP2)	Capability: Development of skills in children and adults
Positive emotions (*n* = 59)	Enthusiasm/excitement/happiness (*n* = 38) Curiosity/anticipation (*n* = 11) Good for children/treat/fun (*n* = 10)	‘He's very excited about the box’ (father of 8‐year‐old SEN child, TP1) ‘The magic of what's going to be in the box each week’ (father of fussy eater aged 9 years, TP1) ‘She is enthusiastic about it [the box], it's the magic of what it is going to be this week’ (mother of 9‐year‐old child, TP1)	Motivation: Positive experience enhances motivation
New/novel (*n* = 49)	Foods/recipes/flavours (*n* = 43) Variety (*n* = 6)	‘He says it's [flavours] like a party in his mouth’ (mother of 2 children, TP2) ‘Because I'm Indian you see, we just make curries & rice you see but this is a different thing like they have a pizza to make, burger & meatballs, pasta’ (mother of 7 children, TP2) ‘Opened a taste palate for myself & my son’ (mother of 14‐year‐old child with autism, TP2) ‘Moroccan meatball with couscous, I would never have made that because it just sounds like a headache, but the fact that it's all measured out for you already you don't really have to think, easy to do after work’ (mother of 9‐year‐old child, TP2)	Opportunity and capability: Increased acceptance due to access to new ideas, foods, flavours, recipes
Experience (*n* = 34)	Enjoyment/like it (*n* = 30) Willing to try (*n* = 4)	‘Was a real fussy eater…found that if she helped prepare the food she's quite willing to try it [the food] & try different things’ (mother of 2 children, TP1) ‘They really enjoy the new recipes and trying out – and all the ingredients are separately organised, so it's easier for us to understand which ingredient to use’ (carer of 2 children, TP1) ‘I've got one pretty fussy twin and he actually tries new things now, which is brilliant. It's the excitement of making stuff together, cooking together. Erm and then if you've gotta make it, you've gotta try it’ (mother of twin 9‐year‐olds, TP1)	Motivation: Positive experience builds motivation
Togetherness (*n* = 34)	Cooking together (*n* = 15) Eating together (*n* = 10) Learning together (*n* = 6) Social (*n* = 3)	‘More of a family affair’ (mother of 2 children, TP1) ‘Do it [cooking] together, it's like a special time for us’ (mother of 9‐year‐old child, TP1) ‘Some days we cook the recipes with a friend, and she was helping us with the food’ (mother of 7‐year‐old child, TP2)	Motivation: Positive experience builds motivation

## 5 Discussion

At both TP, parents/carers consistently indicated that BRITE Box positively impacted several food‐related behaviours in children and families, including eating and cooking together and trying new foods and flavours. In addition, children were reported to be excited by the boxes. This response to BRITE Box contrasts with other approaches to addressing FI such as use of food banks, often associated with shame and embarrassment as well as gratitude among recipients [45, 46, 47, 48]. BRITE Box was specifically constructed to feel like a gift to children and families; something acknowledged by both.

Like food banks, BRITE Box is free to recipients. Although arguably it is less obvious than visiting a food bank, recipients are still visible within schools. Positioning it as a gift enabling families and children to cook and eat together, a gift given directly to children and whose contents are unknown each week until the ‘mystery box’ is opened, resulted in feelings of excitement expressed by almost all interviewees. Schools had wide latitude to decide which children and families would benefit most; in many cases, allocation was on the basis of low income, but it was also given to children with very restricted eating (e.g., due to sensory or other difficulties) and/or poor nutritional intakes. This may have reduced potential stigma of receiving the boxes. In addition, the recipients were unknown to the organisers and volunteers at BRITE Box who were thus distanced from the direct experience of providing charitable food support. In contrast, those managing frontline food support services interact face‐to‐face with clients, and their perceptions, actions and attitudes can have a direct impact on the dignity of the clients’ experience [49]. For whatever reason/s, emotions of recipients were largely positive, suggesting that the scheme was not stigmatising. Linked to this, low levels of embarrassment associated with the scheme were reported at both TPs. Although significantly greater levels of embarrassment at TP2 than TP1 were reported by parents, more parents were neutral about this at TP2 than TP1 (Table 3). Levels of embarrassment overall were low—this statement received a very low level of agreement (6.2%). This may have been contributed to by the ownership schools had over deciding which children and families would receive the boxes—their multiple reasons for doing so may have helped reduce potential stigma. Nonetheless, given the considerable stigma associated with FI, this aspect needs greater exploration in the future.

An impressive range of food‐related behaviours was reportedly impacted positively by BRITE Box. Families reported that they ate together, cooked together and talked about food together more. This matters since family meals are considered beneficial for multiple outcomes including improved nutritional intakes of children and adolescents [25, 50, 51, 52, 53, 54, 55, 56, 57, 58]. Family meals are associated with lower prevalence of disordered eating [59, 60, 61], better mental health [62] and may protect against childhood obesity [63, 64]. The optimal number of meals families should eat together is unclear although it has been suggested that three or more times a week is most beneficial for health [56, 63]. BRITE Box provides one, perhaps two meals a week (depending on family size), which may represent the starting point of eating together for some families. Family meals offer opportunities for social relationships, communications and nourishment unique to each family [65, 66, 67]. Healthy behaviours can be role‐modelled, and children can learn about acceptable social practices around eating and food behaviours [66, 68]. BRITE Box parents described talking together more about food, and time spent preparing and cooking food together also offers opportunities to talk. The work required to provide family meals—so‐called ‘food work’—has cognitive and physical aspects for example decisions about what to eat, and food acquisition and preparation, respectively [69]. This places pressures on parents/carers [70, 71] particularly in relation to societal expectations of what constitutes a healthy acceptable meal—usually one prepared from scratch, with healthy whole ingredients [71, 72]. Some research suggests that parents with limited resources serve less diverse, more child‐oriented meals, albeit while still trying to serve healthy food [73]. In the context of FI, this is an additional pressure and parents described BRITE Box as alleviating some of this, by providing ideas for healthy child‐appropriate meals, supplying ingredients which could be tried by the family without financial risk and enabling children to help.

Acquisition of food skills to plan, select, prepare and consume food may benefit those with FI [74] and is associated with improved dietary quality [75, 76]. Involvement in food preparation was shown to improve intakes and eating patterns in adolescents, specifically improved consumption of fruit and vegetables, better micronutrient intake patterns and more family meals [77]. Involvement of children at an early age may be particularly beneficial, given the importance of early years for nutrition and potential tracking into adolescence [78]. Food preferences and dietary patterns tend to remain stable after the first 3 or 4 years [78, 79]. Repeated exposure to new foods enhances acceptability and the development of taste preferences [80, 81, 82] and has been shown to extend to foods in the same but not other food groups in infants up to 24 months of age [83]. Much research on repeated exposure has focused on infants and younger children and some authors suggest that older children (e.g., aged 6–11 years) may need more exposures than younger children because they have pre‐established food preferences [81]. Therefore, exposure to new fruit and vegetables via BRITE Box would be hypothesised to potentially improve acceptance of other fruit and vegetables, although this has not yet been tested.

Those dealing with FI struggle to access adequate and affordable foods and food waste is a concern [84, 85, 86]. Foods purchased must be acceptable to the family. Qualitative data from BRITE Box parents showed that this was a considerable stress for them, partially alleviated by the boxes. Dietary quality tends to be worse in low‐income groups, with lower intakes of fruit and vegetables and higher intakes of foods often rich in fat, salt and sugar [4, 5, 6] which are cheaper [8, 87, 88]. Analysis of meal kit subscriptions suggests that they have potential to improve vegetable consumption depending on the specific recipes chosen [89]. BRITE Box does not offer a choice but provides the recipe and ingredients for a single meal weekly, all of which include at least two vegetables. It is free to recipients, enabling risk‐free trials of new tastes and textures which can be reproduced cheaply, highlighted by multiple parents/carers as beneficial to themselves and their children.

Beyond exposure to new foods, involving children in their preparation and cooking also increases their acceptability in the community setting [90] and is recommended for skills acquisition from an early age [91]. BRITE Box recipes are designed to be followed by children, under adult supervision. Research suggests that interventions to promote family meals should address sharing responsibility for meal planning and preparation between parents and children [92]. Other research on meal kits demonstrated increased cooking self‐efficacy and cooking techniques following their use [93], and reduced food waste [94]. Home‐delivered meal kits improved diet by encouraging vegetable consumption and appropriate portion sizes, cooking knowledge and skills of parents increased, while children engaged more in meal preparation, although this occurred in older (13–18‐year‐old) rather than younger (6–12‐year‐old) children [95]. However, this scheme differed from BRITE Box; it was a paid subscription not specifically aimed at children. Early involvement of children in food‐related tasks could encourage them to try new foods [96], an aspect of BRITE Box that many parents reported.

Ideally, children and families receiving BRITE Box will continue to cook and eat together. Theories of behaviour change recognise that multiple factors influence behaviours. The COM‐B model is a framework of behaviour change which suggests that behaviour change will occur if **C**apability, **O**pportunity and **M**otivation are addressed [44]. ‘Capability’ in this context is building the confidence and skills to prepare food; ‘opportunity’ is having the chance to try different foods and recipes and to develop skills, and ‘motivation’ is the desire to cook and prepare foods. Parents in this study were clear that both they and their child/ren had increased confidence with cooking and had gained new kitchen skills (Tables 3, 4 and 7). Receiving BRITE Box gave them the opportunity to cook together, and the excitement and enjoyment they experienced from the boxes suggest enhanced motivation. Given this, greater engagement in cooking and eating together, as reported by parents/carers in this study, is unsurprising and several parents reported keeping and reusing favourite recipes. Whether these behaviours will be maintained post BRITE Box remains to be seen, but greater food and kitchen literacy acquired by children is likely to confer longer term benefits. From an educational perspective, many parents/carers reported that the scheme encouraged wider skills acquisition in their children, including measuring and reading. This may also have increased children's confidence and resilience, with possible mental health benefits (although this was not measured).

This evaluation suggests the efficacy of a community‐based meal kit for children and families, carefully constructed as a positive child‐centred intervention. Data are limited to those who completed questionnaires and/or agreed to be interviewed, who may feel particularly strongly about the scheme (positively or negatively). However, our findings are corroborated by teachers and school staff who administer BRITE Box (data not reported). It is also limited by the lack of a control or comparison group. Box is intended to be received for a whole school year but in practice may be administered differently in different schools and the effects of this are unclear. Whether the positive effects of BRITE Box reported here will be maintained in the longer term is also unclear. We are currently evaluating both different administration models (e.g., boxes received by whole classes vs. targeted to some children, and receiving boxes for the whole school year vs. one or two school terms) and exploring longer term effects of the scheme. Although parents/carers reported greater acceptance of diverse foods including vegetables, dietary intakes were not assessed. However, current findings suggest that this meal kit aimed specifically at children has considerable efficacy on current and potentially longer term food‐related behaviours in children and families, worthy of further exploration which should include assessment of dietary intakes.

In conclusion, a child‐centred meal kit administered via schools to low‐income and other vulnerable groups has potential to increase confidence and cooking skills of children and parents/carers, increase acceptability of foods and to improve a range of food‐related behaviours including cooking and eating together.

## Author Contributions

**Hilda Mulrooney:** conceptualisation (equal), formal analysis (equal), project administration (equal), data curation (equal), funding acquisition (equal), visualisation (equal), methodology (equal), writing – original draft preparation (lead), writing – review and editing (equal). **Ronald Ranta:** conceptualisation (equal), funding acquisition (equal), formal analysis (equal), project administration (equal), methodology (equal), data curation (equal), visualisation (lead), writing – review and editing (equal). **Nevena Nancheva:** conceptualisation (equal), methodology (equal), formal analysis (equal), funding acquisition (lead), project administration (equal), data curation (equal), visualisation (equal), writing – review and editing (equal). **Dee Bhakta:** conceptualisation (equal), methodology (equal), data curation (equal), funding acquisition (lead), writing – review and editing (equal). **Sarah Sumpter:** project administration (equal), data curation (equal), writing – review and editing (equal). **Nick Dawson:** conceptualisation (lead), visualisation (equal), writing – review and editing (equal). **Ruth Dawson:** conceptualisation (lead), visualisation (equal), writing – review and editing (equal).

## Ethics Statement

Ethics approval for this work was received from Kingston University Research Ethics Committee. All work was carried out in accordance with the 2013 Declaration of Helsinki.

## Conflicts of Interest

Ruth and Nick Dawson are employed by Voices of Hope, the charity which runs BRITE Box. They codesigned the questionnaires but did not conduct any of the research or analysis of the data. Ronald Ranta is currently a consultant for, and Sarah Sumpter is employed by Voices of Hope; neither was employed there at the time the research was carried out. Hilda Mulrooney volunteers for BRITE Box. None of the researchers were commissioned or paid to carry out this work.

### Peer Review

The peer review history for this article is available at https://www.webofscience.com/api/gateway/wos/peer-review/10.1111/jhn.70038.

## Supporting information

Supporting information.

Supporting information.

## Data Availability

The data that support the findings of this study are available from the corresponding author upon reasonable request.
