# Supplementary material for: Impact of a Recipe Kit Scheme (BRITE Box) on Cooking and Food‐Related Behaviours of Children and Families: Exploring Parental/Carer Views
Source: J Hum Nutr Diet. 2025 Mar 13;38(2):e70038. doi: 10.1111/jhn.70038 (PMC11905342; doi:10.1111/jhn.70038)
Supplement: Supplementary file 1 — Supporting information. [file JHN-38-0-s002.docx]

Appendix 1: Questionnaire

Brite Box evaluation

We would like to understand how Brite Box works, from different perspectives. As a parent/carer, your views will be very helpful. In this survey, we will ask you some questions about yourself/ your family first, and then about how you have found Brite Box. Your feedback is anonymous and all information will be pooled together so no individual can be identified. If you are willing to be interviewed, your contact details will be used ONLY to arrange the interview, and then deleted.

Thank you for your help.

# **About you.**

1. Which option best describes your gender? (woman; man; non-binary; prefer not to say)
2. Which option best describes your ethnicity? (White e.g. British, Irish, any other white background; Black/Black British e.g. Caribbean, African, any other Black background; Asian/Asian British e.g. Indian, Pakistani, Bangladeshi, other Asian background; Mixed e.g. White/Black African/Caribbean, White & Asian, any other; Other ethnic groups e.g. Chinese, any other; prefer not to say)
3. What is your age in years? (<30; 30-39; 40-49; 50-59; 60 and above; prefer not to say)
4. Do you consider yourself to have a disability? (yes; no; prefer not to say)
5. How many children do you have living at home with you?
6. What ages are your children?
7. What school/youth groups do they attend?

**About BRITE Box.**

The next set of questions are about your experience of BRITE Box. There are no right or wrong answers – please say what you think.

1. How long have you been receiving BRITE Box? (0-3 months; 3-6 months; >6 months)
2. Thinking about you and your family, please tick ONE option for EACH of the statements below:

|  | Strongly  disagree | Disagree | Neither agree nor disagreed | Agree | Strongly agree |
| --- | --- | --- | --- | --- | --- |
| My child/ren are excited when we get a BRITE Box |  |  |  |  |  |
| My child/ren are embarrassed by getting BRITE Box |  |  |  |  |  |
| We have tried new foods since starting BRITE Box |  |  |  |  |  |
| BRITE Box has introduced us to new flavours |  |  |  |  |  |
| We have cooked together more than before since starting BRITE Box |  |  |  |  |  |
| We have learnt new & different kitchen skills since starting BRITE Box |  |  |  |  |  |
| We eat together as a family more since starting BRITE Box |  |  |  |  |  |
| We talk more about food since starting BRITE Box |  |  |  |  |  |
| My child/ren have gained confidence with cooking since we started BRITE Box |  |  |  |  |  |
| We use more leftovers more since starting BRITE Box |  |  |  |  |  |
| We eat more vegetables since starting BRITE Box |  |  |  |  |  |
| We always follow the BRITE Box recipe |  |  |  |  |  |
| We never use the BRITE Box recipe; we use the foods to make other dishes |  |  |  |  |  |
| We eat more healthy foods because of BRITE Box |  |  |  |  |  |
| We will use some of the BRITE Box recipes again |  |  |  |  |  |
| My child/ren have tried new foods because they have been involved in preparing & cooking them |  |  |  |  |  |
| Overall our experience of BRITE Box has been positive |  |  |  |  |  |
|  |  |  |  |  |  |

1. Can you tell us more about new foods/flavours you have tried since starting BRITE Box? Can you give some examples? Leave blank if this does not apply to you.
2. Which of the following skills have your child/ren gained since starting BRITE Box? Tick **ALL** that apply (knife skills; preparing vegetables; working with meat/chicken; making sauces; frying/stir frying; working with herbs/spices; cooking rice/potatoes/cous cous/pasta; using the oven; using the hob; none, my child/ren already had those skills; other
3. If you ticked ‘other’ to the question above, can you tell us what skills your child/ren have gained since starting BRITE Box?
4. Please tick ONE option for each of the following statements, in relation to you and your family:

|  | Strongly disagree | Disagree | Neither agree not disagree | Agree | Strongly agree |
| --- | --- | --- | --- | --- | --- |
| There are foods we would like to eat but cannot afford to buy |  |  |  |  |  |
| I would be embarrassed to use a food bank |  |  |  |  |  |
| BRITE Box helps me with my food budget |  |  |  |  |  |
| I am more confident in the kitchen since starting BRITE Box |  |  |  |  |  |
| My child/ren are more confident about food/cooking since starting BRITE Box |  |  |  |  |  |
| I have found the Chef Booklet useful |  |  |  |  |  |
| Taking part in BRITE Box has improved our mental wellbeing |  |  |  |  |  |

1. What do you think are the biggest changes in your child/ren since starting BRITE Box? Leave blank if you do not think they have changed.
2. What do you like LEAST about BRITE Box?
3. What do you like MOST about BRITE Box?
4. What are your family’s top three BRITE Box recipes? Can you explained what they liked about those recipes?
5. Is there anything else you would like to say about your participation in BRITE Box?
6. Could you help us by taking part in an optional interview about your experiences with BRITE Box? If so, please leave your email or phone number below (for the purposes of organising the meeting only; it will then be destroyed). The interview can be held online, on the phone or face-to-face, whatever you prefer.
7. In the past have you used any other form of food support e.g. food bank, social supermarket? (yes; no)
8. If so, what other sort of food support have you used? How did you find it?
