# Supplementary material for: Impact of a Recipe Kit Scheme (BRITE Box) on Cooking and Food‐Related Behaviours of Children and Families: Exploring Parental/Carer Views
Source: J Hum Nutr Diet. 2025 Mar 13;38(2):e70038. doi: 10.1111/jhn.70038 (PMC11905342; doi:10.1111/jhn.70038)
Supplement: Supplementary file 2 — Supporting information. [file JHN-38-0-s001.docx]

***Appendix 2: Interview guide for parents/carers.***

**Individual** **outline questions for parents/carers**

1. Background context: how long have you been getting BRITE Box, numbers/ages of children.
2. What led up to your involvement with BRITE Box? How did you hear about it?
3. Can you talk me through how BRITE Box works for you?
4. How does the family react when you get a new BRITE Box?
5. Have you/family tried new foods/recipes/ tastes since starting BRITE Box? Tell me about those.
6. Do you think your/children’s relationships with food have changed since starting BRITE Box? For example, have mealtimes changed in any way?
7. Have you/family gained any new skills since starting BRITE Box? Can you talk me through them?
8. Have you used other food support initiatives, like the food bank? (if so, can you tell me about how you found them?)
9. How do you manage when your food budget is tight?
10. What is the value of BRITE Box, in your opinion? What is the best thing about it?
11. What has the impact of BRITE Box been for you/your family?
12. Do you think that BRITE Box has impacted upon the wellbeing of your family? Can you tell me more?
13. What do you think are the causes of food poverty in this country?
14. How do you think it should be addressed?
15. Is there anything else you would like to add?

**Thank you for your time.**
